# Wound Closure Promotion by Leucine-Based Pseudo-Proteins: An In Vitro Study

**DOI:** 10.3390/ijms25179641

**Published:** 2024-09-06

**Authors:** Mariam Ksovreli, Tinatin Kachlishvili, Mariam Skhvitaridze, Lili Nadaraia, Rusudan Goliadze, Luka Kamashidze, Knarita Zurabiani, Tatuli Batsatsashvili, Nino Kvachantiradze, Marekhi Gverdtsiteli, Temur Kantaria, Olivier Piot, Marie-Pierre Courageot, Christine Terryn, Pavel Tchelidze, Ramaz Katsarava, Nina Kulikova

**Affiliations:** 1 Institute of Cellular and Molecular Biology, Agricultural University of Georgia, 0159 Tbilisi, Georgia; m.ksovreli@agruni.edu.ge (M.K.); t.kachlishvili@agruni.edu.ge (T.K.); mskhv2020@agruni.edu.ge (M.S.); rgoli2018@agruni.edu.ge (R.G.); lkama2017@agruni.edu.ge (L.K.); kzura2019@agruni.edu.ge (K.Z.); tbats2019@agruni.edu.ge (T.B.); nkvac2020@agruni.edu.ge (N.K.); 2 Carl Zeiss Scientific and Education Center, New Vision University, 0159 Tbilisi, Georgia; lnadaraia@newvision.ge; 3 Institute of Chemistry and Molecular Engineering, Agricultural University of Georgia, 0159 Tbilisi, Georgia; m.gverdtsiteli@agruni.edu.ge (M.G.); te.kantaria@agruni.edu.ge (T.K.); r.katsarava@agruni.edu.ge (R.K.); 4 BioSpectroscopie Translationnelle (BioSpecT) Unit, University of Reims Champagne-Ardenne, 51100 Reims, France; olivier.piot@univ-reims.fr (O.P.); marie-pierre.courageot@univ-reims.fr (M.-P.C.); 5 La plateforme en Imagerie Cellulaire et Tissulaire (PICT), University of Reims Champagne-Ardenne, 51100 Reims, France; christine.terryn@univ-reims.fr; 6 Faculty of Healthcare, East European University, 0159 Tbilisi, Georgia; pavle.tchelidze@eeu.edu.ge

**Keywords:** leucine-based pseudo-proteins (LPPs), wound healing, cell proliferation and migration, RAW264.7, primary mouse skin fibroblasts

## Abstract

Our research explores leucine-based pseudo-proteins (LPPs) for advanced wound dressings, focusing on their effects on wound healing in an in vitro model. We assessed three types of LPP films for their ability to enhance wound closure rates and modulate cytokine production. They all significantly improved wound closure compared to traditional methods, with the 8L6 and copolymer films showing the most pronounced effects. Notably, the latter exhibited an optimal cytokine profile: an initial burst of pro-inflammatory TNF-α, followed by a controlled release of IL-6 during the proliferative phase and a significant increase in anti-inflammatory IL-10 during remodeling. This balanced cytokine response suggests that the copolymer film not only accelerates wound closure but also supports a well-regulated healing process, potentially reducing fibrosis and abnormal scarring, underscoring the potential of copolymer LPPs as advanced wound dressing materials. Future research will aim to elucidate the specific signaling pathways activated by the copolymer LPP to better understand its mechanism of action. Overall, LPP films offer a promising approach to improving wound care and could lead to more effective treatments for complex wounds.

## 1. Introduction

Wounds are a significant problem for healthcare systems due to their susceptibility to infection, the prevalence of chronic and non-healing types, the high costs of long-term care, and the specialized care that they require [1]. Effective wound management is essential to overcoming these challenges, improving patient outcomes, and reducing the overall burden on healthcare resources. Therefore, the development of wound dressings that can promote a proper wound healing process, ensuring normal function restoration in the repaired tissue, is a question of paramount importance [1,2,3].

The limitations of existing wound dressings and the unmet needs of patients with complex or non-healing wounds are critical aspects of advancing medical care. Materials that promote faster wound closure can reduce the overall healing time, benefiting both patients and healthcare systems. Novel materials can be tailored to suit various types of wounds (e.g., acute, chronic, diabetic ulcers, burns) and different stages of the healing process. Materials designed to be biocompatible minimize the risk of adverse reactions and promote natural tissue integration [1,2,3].

Our research explores the potential of the relatively novel leucine-based pseudo-proteins (LPPs) [4,5] as advanced biocompatible materials for wound dressings.

Recently, we demonstrated the excellent cell-supporting properties of polymeric films prepared from three different biomimetic LPPs: 1L6 (composed of carbonic acid, L-leucine, and 1,6-hexanediol), 8L6 (composed of sebacic acid, L-leucine, and 1,6-hexanediol), and a copolymer (CoP) constituted of 70 mol% 1L6 and 30 mol% 8L6 [6]. The properties of the LPP films were tested using two cell types—primary mouse skin fibroblasts and murine monocyte/macrophage RAW264.7 cells—which both showed prominent adhesion and optimal cell spreading. An analysis of actin cytoskeleton organization revealed numerous motility-associated structures in both cell types, and while all three LPP films stimulated cell proliferation, the extent varied among them. Additionally, two LPP films (1L6 and CoP) promoted macrophage migration.

Considering our investigation of the LPP films’ surface topography, which revealed sub-micro differences between the three [6], we hypothesized that these might account for the varied effects on cell physiology. Overall, the ability of the studied LPP films to provide highly adhesive support and stimulate cell migration and proliferation provided a strong basis for suggesting their potential use as wound dressings, as enhanced cell migration and adhesion can accelerate wound closure due to cells’ more efficient movement towards the wound site.

Different surface topographies can influence not only cell adhesion strength, migration patterns, and proliferation rates [7,8,9,10,11,12,13], but also modulate cytokine expression, affected by the cellular microenvironment, including surface topography. Variations in topography could lead to differences in the mechanical stress experienced by cells, thereby altering the cytokine profiles [9,10]. This modulation can result in either a pro-inflammatory or anti-inflammatory response, impacting various wound healing stages such as inflammation, proliferation, and remodeling [14,15,16,17,18,19].

To explore the wound healing potential of the studied LPP films, we evaluated not only their influence on wound closure in an in vitro wound healing model [20,21], but also their effects on the cytokine secretion profile in cells grown on their surface, considering that wound closure is carefully orchestrated by the cytokines released at the wound site [14,15,16,17,18,19]. Two types of cells were used in this study, based on their role in the wound healing process: macrophages (RAW264.7) and fibroblasts (primary mouse skin fibroblasts—pMSFs). The interaction between these two types of cells is pivotal for the effective performance of the wound healing process [18].

The results of our research clearly demonstrated that LPP films, especially 8L6 and CoP, significantly enhance wound closure rates compared to traditional collagen-based controls, supporting our hypothesis that these films provide a more complex and conducive extracellular environment for wound healing. Their superior performance in accelerating wound closure highlights their potential as innovative wound care materials, offering promising opportunities for improving wound management and patient outcomes. The unique properties of these films, particularly their ability to dynamically modulate the wound healing process through cytokine regulation without the addition of any immunomodulatory agents, represent a significant advancement in the development of next-generation wound dressings. Further research will be essential to fully realize the clinical benefits of these advanced biomaterials.

## 2. Results

### 2.1. In Vitro Wound Healing Assay

To evaluate fibroblasts’ ability to migrate to a wound area, a scratch assay is commonly used: a cell monolayer is “wounded” by creating a scratch, and, afterwards, the dynamics of re-seeding the gap are monitored by bright-field phase-contrast imaging. Wound closure happens when cell–cell contacts are reestablished and no denuded space is left.

**Figure 1:**
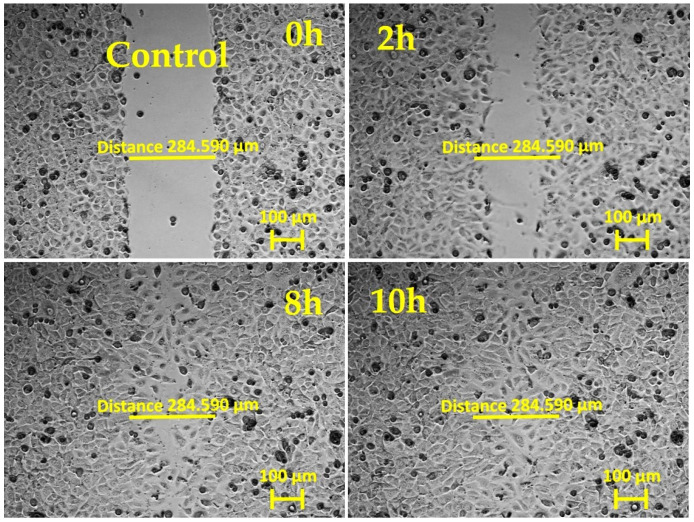
In vitro “wound” healing model. Primary mouse skin fibroblasts on a glass slide (after 24 h of incubation). After creating the “scratch”, the time-lapse imaging was performed every 10 min for 20 h using the inverted phase-contrast microscopy mode. The presented images are frames cut from the recordings and taken 0, 2, 8, and 10 h after scratching. The initial “wound” width (distance across the scratch) was 289.301 μm, measured with Zen 3.0 software.

**Figure 2:**
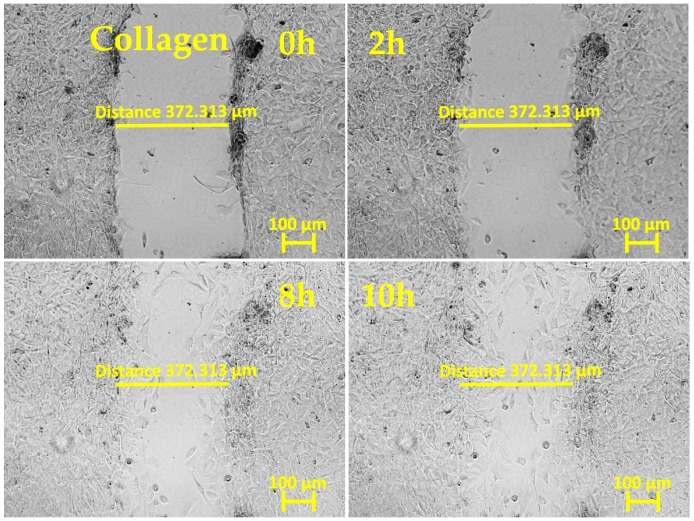
In vitro “wound” healing model. Primary mouse skin fibroblasts on a collagen-covered slide (after 24 h of incubation). After creating the “scratch”, the time-lapse imaging was performed every 10 min for 20 h using the inverted phase-contrast microscopy mode. The presented images are frames cut from the recordings and taken 0, 2, 8, and 10 h after scratching. The initial ‘wound’ width (distance across the scratch) was 372.313 μm, measured with Zen 3.0 software.

**Figure 3:**
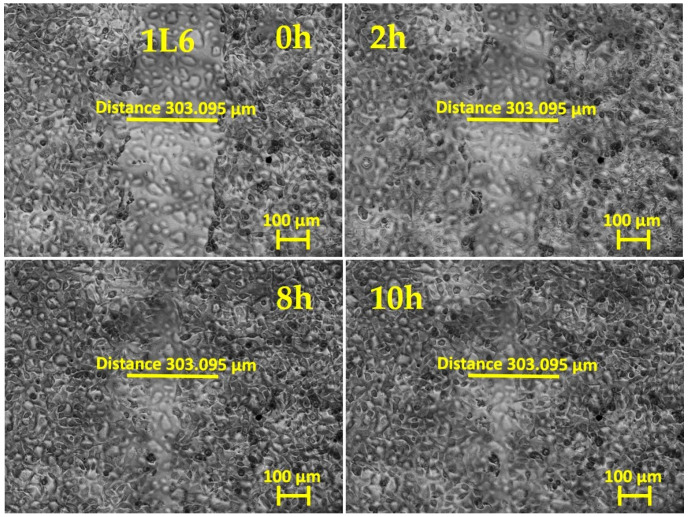
In vitro “wound” healing model. Primary mouse skin fibroblasts on a 1L6 LPP film-covered slide (after 24 h of incubation). After creating the “scratch”, the time-lapse imaging was performed every 10 min for 20 h using the inverted phase-contrast microscopy mode. The presented images are frames cut from the recordings and taken 0, 2, 8, and 10 h after scratching. The initial ‘wound’ width (distance across the scratch) was 303.095 μm, measured with Zen 3.0 software.

**Figure 4:**
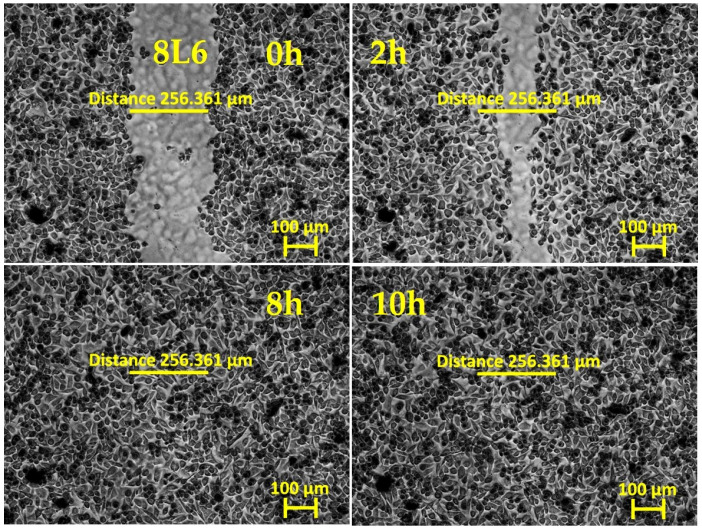
In vitro “wound” healing model. Primary mouse skin fibroblasts on an 8L6 LPP film-covered slide (after 24 h of incubation). After creating the “scratch”, the time-lapse imaging was performed every 10 min for 20 h using the inverted phase-contrast microscopy mode. The presented images are frames cut from the recordings and taken 0, 2, 8, and 10 h after scratching. The initial ‘wound’ width (distance across the scratch) was 254.408 μm, measured with Zen 3.0.

**Figure 5:**
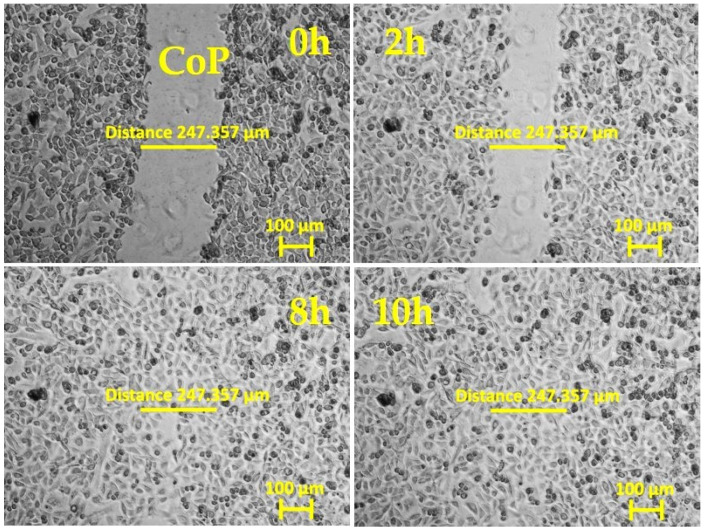
In vitro “wound” healing model. Primary mouse skin fibroblasts on a CoP LPP film-covered slide (after 24 h of incubation). After creating the “scratch”, the time-lapse imaging was performed every 10 min for 20 h using the inverted phase-contrast microscopy mode. The presented images are frames cut from the recordings and taken 0, 2, 8, and 10 h after scratching. The initial ‘wound’ width (distance across the scratch) was 254.449 μm, measured with Zen 3.0.

In our experiments, primary mouse skin fibroblasts (pMSFs) were seeded until a confluent layer was achieved on untreated glass slides or slides covered with LPP films or collagen. The samples were then scratched and placed under an inverted phase-contrast microscope (Carl Zeiss AG, Oberkochen, Germany) for 20 h of live imaging. Figure 1, Figure 2, Figure 3, Figure 4 and Figure 5 present images depicting the dynamics of wound closure for the control, collagen, 1L6 LPP film, 8L6 LPP film, and CoP LPP film, respectively. In each figure, pictures taken at four time points are presented: 0, 2, 8, and 10 h. Filming was performed for 20 h for each sample, but, as it is clearly visible from Figure 1, Figure 2, Figure 3, Figure 4 and Figure 5, after 10 h, the “wound” was already closed in all instances. For each sample, the speed of wound closure was calculated based on the initial width between the wound edges and the exact time of gap repopulation. 

**Figure 6:**
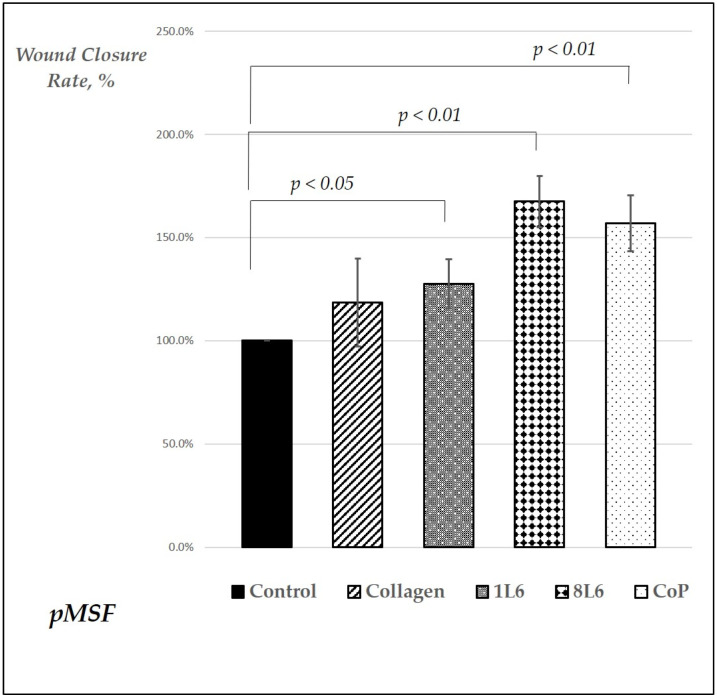
Wound closure rate for pMSFs. The data are presented as a percentage relative to the control (control set as 100%), with three as the minimum number of experiments in each case. The data are shown as mean values (*n* = 3–5), with error bars representing the standard error of mean (SEM). Statistically significant differences compared to the control are indicated by the corresponding *p*-values displayed above the bars.

The wound closure speed data for all five samples are presented in Figure 6. For each sample type, at least three independent experiments were performed. According to the results, the wound closure speed for all LPP films exceeded the closing speed in the control condition. For the 8L6 and CoP LPP films, the wound closure rate was even superior to that of samples grown on collagen, a common component of the extracellular matrix (ECM), known for creating favorable cell adhesion and migration conditions. 

The full video files for all five samples are provided as Supplementary Materials (Videos S1–S5).

### 2.2. Evaluation of Secreted Cytokine Levels

In cultures of both RAW264.7 cells and pMSFs grown on LPP films, we assessed the levels of key cytokines involved in various wound healing stages: TNF-α, a pro-inflammatory cytokine critical to the inflammatory stage; IL-6, a pleiotropic cytokine involved in the proliferative stage; and IL-10, an anti-inflammatory cytokine regulating the remodeling stage.

**Figure 7:**
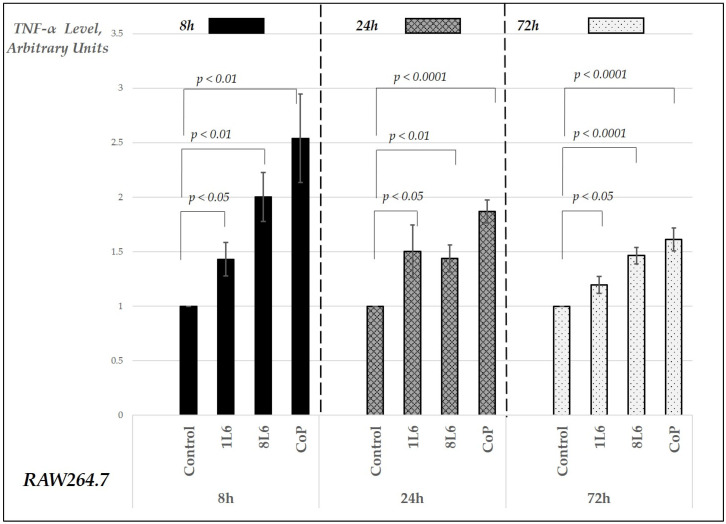
Secreted TNF-α levels in RAW264.7 cell cultures grown on LPP films (1L6, 8L6, and CoP) after 8, 24, and 72 h of incubation. TNF-α levels are expressed in arbitrary units relative to the control (set to 1) and grouped by incubation time. Data represent mean values from a minimum of three experiments (*n* = 3–6), with error bars indicating the mean (SEM). Statistically significant differences compared to the control are indicated by the corresponding *p*-values displayed above the bars.

**Figure 8:**
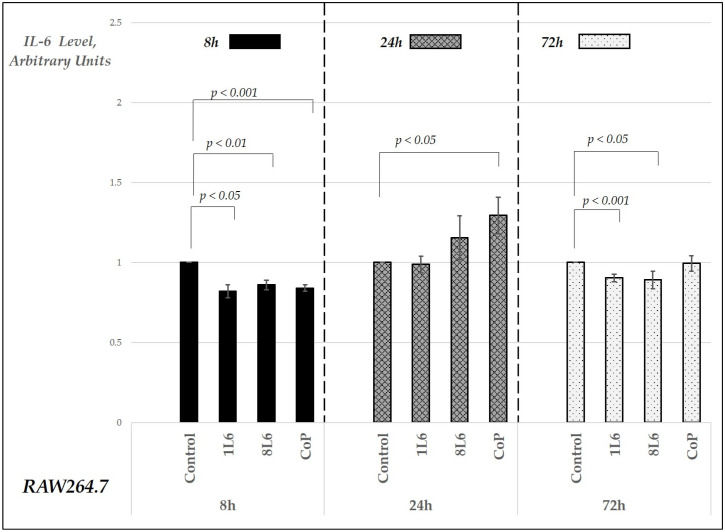
Secreted IL-6 levels in RAW264.7 cell cultures grown on LPP films (1L6, 8L6, and CoP), measured at 8, 24, and 72 h of incubation. IL-6 levels are expressed in arbitrary units relative to the control (set to 1) and grouped by incubation time. Data represent mean values from a minimum of three experiments (*n* = 3–6), with error bars indicating the mean (SEM). Statistically significant differences compared to the control are indicated by the corresponding *p*-values displayed above the bars.

**Figure 9:**
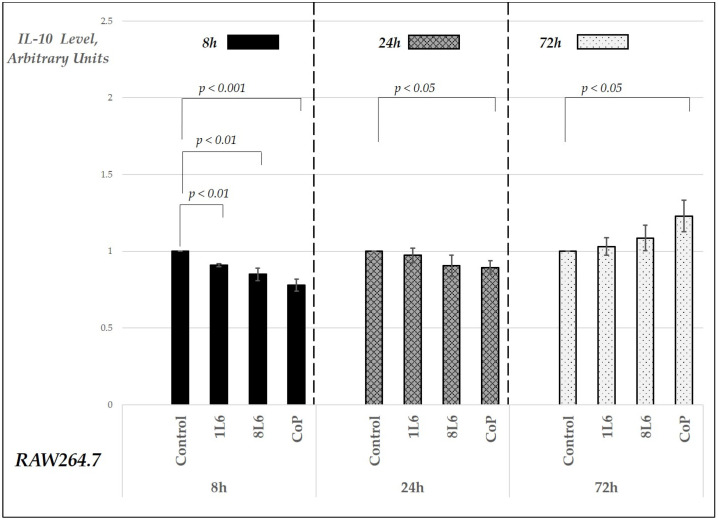
Secreted IL-10 levels in RAW264.7 cell cultures grown on LPP films (1L6, 8L6, and CoP), measured at 8, 24, and 72 h of incubation. IL-10 levels are expressed in arbitrary units relative to the control (set to 1) and grouped by incubation time. Data represent mean values from a minimum of three experiments (*n* = 3–6), with error bars indicating the mean (SEM). Statistically significant differences compared to the control are indicated by the corresponding *p*-values displayed above the bars.

In the RAW264.7 macrophage cultures, the secreted cytokine levels were measured in cell culture supernatants at three different time points—8, 24, and 72 h of incubation. The chosen time points—8 h, 24 h, and 72 h—were selected to capture key phases of the wound healing process, which typically progresses through different stages over time. At 8 h, the early cellular response, including initial migration and proliferation, can be observed. By 24 h, these processes become more established, and significant changes in cytokine levels and cellular activity are expected. The 72 h mark allows for the observation of later stages, including tissue remodeling and the resolution of inflammation. This selection of time points provides a comprehensive view of the wound healing dynamics at critical intervals. In the cell cultures grown either in control conditions (on plastic, on 96-well plates) or on LPP films (on 96-well plates pre-covered with LPPs), the supernatants were collected and assayed for their TNF-α, IL-6, and IL-10 levels using the sandwich enzyme-linked immunosorbent assay (ELISA) method; the results are presented in Figure 7, Figure 8 and Figure 9, respectively, in arbitrary units relative to the control, set to 1.

Our findings indicate that RAW264.7 macrophage cultures grown on LPP films (1L6, 8L6, and CoP) exhibited a significant increase in the secreted TNF-α levels compared to the control for all three types of LPP films (Figure 7). This elevation was noted at 8, 24, and 72 h of incubation, with the most pronounced one occurring at the earliest time point and noticeable decreases later on compared to the initial surge in TNF-α production. For IL-6, a decrease was detected for all three LPP films at the 8 h time point, while, after 24 h, a significant increase was registered for the CoP film (Figure 8).

Regarding the IL-10 levels, a decrease was observed across all three LPP films at the 8 h time point, similar to the trend seen with IL-6. After 24 h, only the CoP film exhibited a statistically significant reduction in IL-10 levels (Figure 9), and, by 72 h, the IL-10 secretion levels had increased for all three films compared to the 24 h levels. However, this increase was most pronounced and statistically significant only in the CoP film-grown cell cultures (Figure 9).

**Figure 10:**
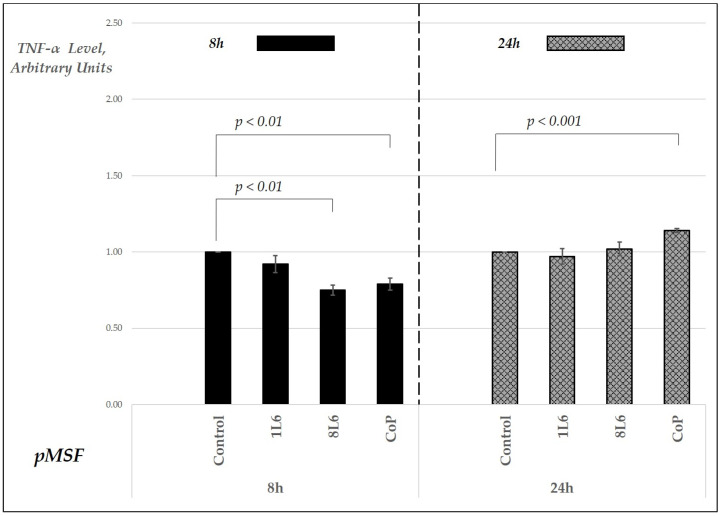
Secreted TNF-α levels in pMSF cell cultures grown on LPP films (1L6, 8L6, and CoP), measured at 8 and 24 h of incubation. TNF-α levels are expressed in arbitrary units relative to the control (set to 1) and grouped by incubation time. Data represent mean values from a minimum of three experiments (*n* = 3–6), with error bars indicating the mean (SEM). Statistically significant differences compared to the control are indicated by the corresponding *p*-values displayed above the bars.

**Figure 11:**
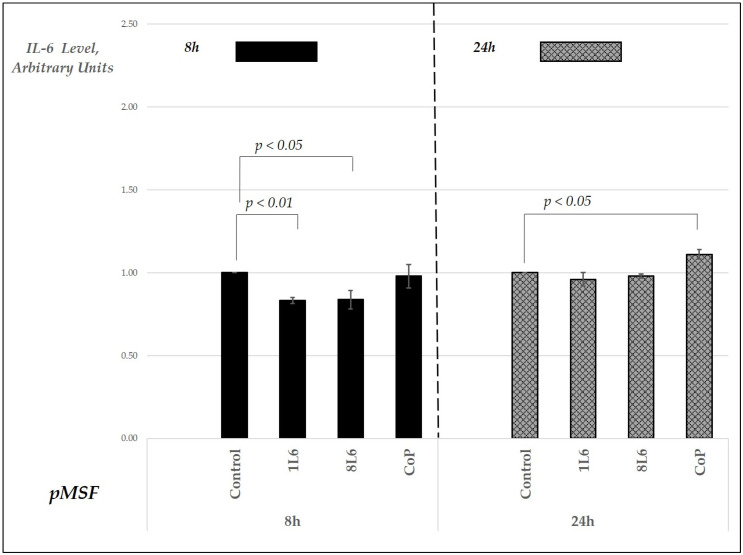
Secreted IL-6 levels in pMSF cell cultures grown on LPP films (1L6, 8L6, and CoP), measured at 8 and 24 h of incubation. IL-6 levels are expressed in arbitrary units relative to the control (set to 1) and grouped by incubation time. Data represent mean values from a minimum of three experiments (*n* = 3–6), with error bars indicating the mean (SEM). Statistically significant differences compared to the control are indicated by the corresponding *p*-values displayed above the bars.

**Figure 12:**
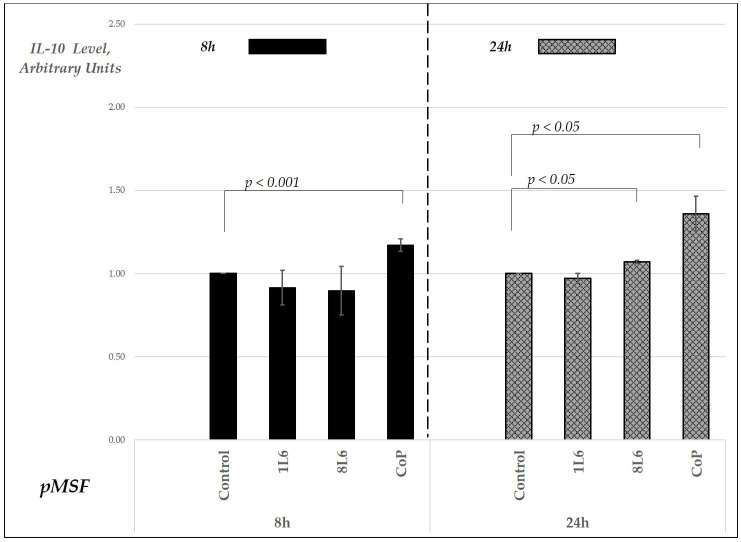
Secreted IL-10 levels in pMSF cell cultures grown on LPP films (1L6, 8L6, and CoP), measured at 8 and 24 h of incubation. IL-10 levels are expressed in arbitrary units relative to the control (set to 1) and grouped by incubation time. Data represent mean values from a minimum of three experiments (*n* = 3–6), with error bars indicating the mean (SEM). Statistically significant differences compared to the control are indicated by the corresponding *p*-values displayed above the bars.

In the pMSF cultures grown either under control conditions (on plastic, on 96-well plates) or on LPP films (on 96-well plates pre-covered with LPPs), the secreted cytokine levels were measured in the cell culture supernatants after 8 and 24 h of incubation. The TNF-α, IL-6, and IL-10 levels were assayed using the sandwich enzyme-linked immunosorbent assay (ELISA) method; the results are shown in Figure 10, Figure 11 and Figure 12, respectively, in arbitrary units relative to the control, set to 1.

According to the results, in the pMSF cell cultures, the TNF-α level decreased in the LLP film-grown samples at the 8 h time point (Figure 10), while an increase was detected only in the CoP film-grown samples after 24 h (Figure 10). For IL-6, a decrease was noted at the 8 h time point in the 1L6 and 8L6 LPP film-grown fibroblast cultures. By 24 h, the IL-6 levels in these cultures were similar to the control. In contrast, the IL-6 levels in the samples grown on CoP were comparable to the control at the 8 h point but showed an increase after 24 h of incubation (Figure 11).

In terms of IL-10 secretion in fibroblast cultures, only the CoP film induced an increase in the levels of this cytokine, which instead remained similar to the control in the cultures grown on the other two films at both time points (Figure 12).

### 2.3. Macrophage Phenotyping

**Figure 13:**
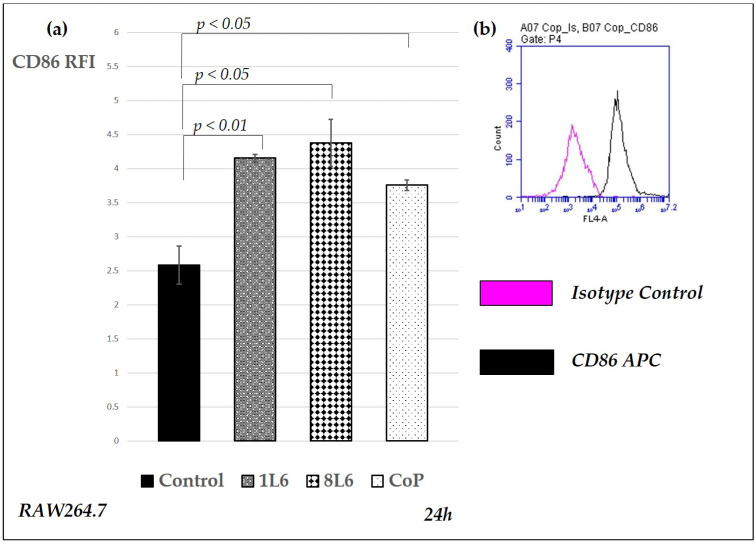
Surface expression of CD86 in RAW264.7 cells grown on control and LPP films (1L6, 8L6, and CoP). CD86 expression was evaluated by flow cytometry (BD Accuri C6) after 24 h of incubation. (**a**) On the Y axis, the relative fluorescence intensity (RFI) = mean fluorescence intensity (MFI) of CD86-APC/MFI of the isotype control is shown. The minimal number of experiments in each case was four. The columns show the mean (*n* = 10–13), while the bars show the mean (SEM). Statistically significant differences compared to the control are indicated by the corresponding *p*-values displayed above the bars. (**b**) Representative histogram overlay showing isotype control and CD86 expression in CoP-grown cell cultures. Magenta line: Isotype control; black line: CD86 APC.

**Figure 14:**
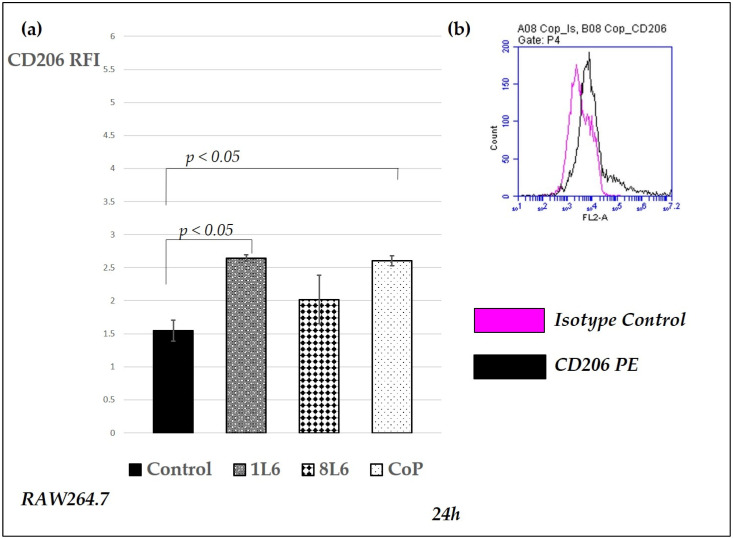
Surface expression of CD206 in RAW264.7 cells grown on control and LPP films (1L6, 8L6, and CoP). CD206 expression was evaluated by flow cytometry (BD Accuri C6) after 24 h of incubation. (**a**) On the Y axis, the relative fluorescence intensity (RFI) = mean fluorescence intensity (MFI) of CD206-PE/MFI of the isotype control is shown. The columns show the mean (*n* = 5–8), while the bars show the mean (SEM). Statistically significant differences compared to the control are indicated by the corresponding *p*-values displayed above the bars. (**b**) Representative histogram overlay showing isotype control and CD206 expression in CoP-grown cell cultures. Magenta line: Isotype control; black line: CD206 PE.

To evaluate the activation status of control and LPP film-grown macrophages, we studied the surface expression of CD86 and CD206 in RAW264.7 cells. Elevated CD86 expression is typical for pro-inflammatory M1 macrophages, while CD206 is indicative of alternatively activated macrophages. Phenotyping was performed using subsequent monoclonal antibodies directly fluorescent-conjugated to murine CD86 and CD206, while sample analyses were carried out by flow cytometry (BD Accuri C6), whereby the data were expressed in relative fluorescence intensity (RFI), calculated by dividing the mean fluorescence intensity of the sample by that of the control. The CD86 and CD206 expression data are presented in Figure 13 and Figure 14, respectively.

The results show that, at the 24 h time point, CD86 expression was enhanced for all LPP films (Figure 13), corresponding well to the secreted TNF-α levels, as they were both elevated during macrophage activation. Interestingly, CD206 surface expression, which is usually associated with pro-regulatory processes, was also enhanced for 1L6 and CoP, with the highest level detected for cells grown on the latter (Figure 14). 

## 3. Discussion

In recent years, numerous studies have focused on developing novel advanced wound dressings with immunomodulatory properties [22,23,24,25], proposing a wide range of natural and synthetic biocompatible materials and various approaches for endowing them with the desired properties, including the addition of certain soluble factors, antibodies, mixtures of skin-derived factors, embryonic fibroblasts, etc. [22]. Some of these are less cost-effective, while others involve the use of human-derived materials, raising both ethical issues and concerns about disease transmission. Although some of these materials are already undergoing pre-clinical studies, the development of low-cost, easy-to-use, and effective wound dressings remains a critical need for the global healthcare system [1,22,23,24,25].

In our first research step, we evaluated the LPP effects on wound closure using an in vitro wound healing model, hypothesizing that the leucine-based pseudo-proteinic films would provide a more complex extracellular environment with ECM-like properties that could modulate cellular behavior. Therefore, we used cells seeded on collagen-covered plastic—a common ECM component—as an additional control [26,27,28].

The results showed that all three LPP films significantly accelerated wound closure compared to the control conditions (Figure 1, Figure 2, Figure 3, Figure 4, Figure 5 and Figure 6). Two LPP films, 8L6 and CoP, showed closure speeds higher than the collagen-grown cultures, with the highest rate observed on the 8L6 film (Figure 6). This indicates that the studied LPPs promoted both cell migration and proliferation, which aligns with our previous findings [6]. The enhanced wound closure observed on the LPP films compared to the collagen control underscores the importance of these films’ biomimetic properties in facilitating cellular processes essential for wound healing. The differences observed across the samples highlight the differential effects of LPPs, possibly attributed to sub-micro dissimilarities in their surface topography, as shown previously [6].

Although the 8L6 LPP film demonstrated superior properties in accelerating wound closure, we are concerned that such rapid wound healing might have drawbacks, as excessive proliferation at the wound site can lead to fibrosis and abnormal scarring [14,15,16,17,18,19]. Therefore, the moderate acceleration observed with CoP appears optimal, positioning this LPP as the leading candidate for a novel wound dressing material among the three.

While the promotion of cell proliferation and migration is crucial for successful wound closure, it is important to emphasize that this process generally depends on the balance between the pro-inflammatory and regulatory cytokines at the wound site [16,17]. Therefore, evaluating the wound healing-promoting properties of any potential wound dressing material requires assessing its effect on cytokine production by the cell populations involved in wound closure. Thus, while examining the beneficial effects of the LPP films on wound healing, we also measured the levels of key cytokines involved in different stages of this process.

Wound healing requires a careful balance of pro- and anti-inflammatory events: initial acute inflammation is crucial for the healing process, while prolonged chronic inflammation is a main cause of tissue fibrosis and abnormal scarring [16,17]. Therefore, a beneficial cytokine secretion pattern for wound healing would involve an increase in pro-inflammatory cytokines during the initial phase, an elevation in the IL-6 levels needed for the proliferation phase at a later time point, and, finally, after several days, an increase in pro-regulatory cytokines to combat inflammation and promote tissue remodeling [14,15,16,17,18,19].

According to our results, the highest initial burst in TNF-α production was detected for the CoP film; however, this film showed a dramatic decrease of more than 50% after 72 h compared to the 8 h increase (Figure 7). Given that TNF-α, a pro-inflammatory cytokine, plays an important role in the initial phase of wound healing [15], an increase in this cytokine’s level at the 8 h time point is crucial for wound closure promotion. Therefore, the secretion pattern for the CoP film, with a rapid increase followed by a profound decrease, seems optimal for wound healing. Notably, for pMSFs, a TNF-α level decrease was registered for all LPPs at the 8 h time point. Considering the stimulation of this cytokine’s production by macrophages at the same time point, this cell-specific action is advantageous for the wound healing process as it ensures no overwhelming increase in the TNF-α levels at the wound site.

The CoP LPP’s superior wound healing properties were further supported by analyzing the IL-6 and IL-10 production dynamics in cell cultures grown on LPPs (Figure 8 and Figure 9). The IL-6 secretion pattern in CoP film-grown macrophages can be considered optimal: a small burst in IL-6 at the 24 h time point, followed by normalization after 72 h (Figure 8). While this cytokine is crucial during the second, proliferative stage of the wound healing process [19], optimal wound closure should not cause fibrosis, which can occur if extensive uncontrolled fibroblast proliferation continues. Therefore, it is important that the IL-6 levels do not remain high for an extended period.

In the case of IL-10, a cytokine important during the final remodeling stage of wound healing [16,17], a distinguished wound healing-promoting pattern of production was detected in the CoP LPP. This LPP film was the only one to induce an IL-10 level increase at the 72 h time point in both macrophages and fibroblasts (Figure 9 and Figure 12). These data were further supported by the CD206 expression pattern: this molecule’s surface expression, typical for alternatively activated M2 macrophages, was enhanced in the cells grown on CoP (Figure 14).

Overall, our results suggest that, among the studied LPP films, the CoP is the most promising as a wound dressing material, as it not only promotes wound closure but also modulates cytokine production in a way that supports optimal wound healing. This finding aligns with our previous data, which showed that the CoP LPP stimulated both fibroblast and macrophage proliferation at the 8 h and 24 h time points and promoted macrophage migration, making it the superior film in terms of stimulatory properties [6].

Given that all the pseudo-proteinic films used in our experiments were leucine-based and had very similar chemical compositions, with CoP consisting of (1L6)_0.7_-(8L6)_0.3_ [18], the differing cellular responses were likely due to the specific structural properties of each film type and their interaction with the cellular signaling pathways. We have previously shown that all three LPP films differ in terms of porosity and pore size at the sub-micro level [6]. Therefore, we hereby speculate that the differences in these films’ actions could also be attributed to their distinct surface topologies.

Multiple studies have demonstrated that nano/sub-micro-level surface topological properties, such as roughness and porosity, influence the physiology of cells grown on these surfaces by modulating the adhesive complexes regulating cell cycle entry, cell shape, proliferation, and other cellular functions [6,7,8,9,10,11,12,13]. LPP films’ surface topologies can also affect the cytokine expression profiles of macrophages and fibroblasts through various cellular signaling pathways and mechano-transduction mechanisms [29,30,31,32].

Integrins, the primary transmembrane receptors facilitating cell–extracellular matrix (ECM) adhesion, play a central role in this process. Different surface topographies can modulate integrin clustering and activation, impacting downstream signaling pathways [33,34,35,36]. Changes in integrin signaling can influence the activation of focal adhesion kinase (FAK) [34], which subsequently activates various downstream pathways, including MAPK/ERK [35] and NF-κB [36], crucial for cytokine production.

FAK is a critical mediator of signal transduction from integrins and other ECM receptors whose activation leads to the phosphorylation of the downstream targets involved in cell survival, proliferation, and migration. FAK activation has also been shown to regulate the production of cytokines such as TNF-α, IL-6, and IL-10 [34].

The mitogen-activated protein kinase (MAPK) pathway, including extracellular signal-regulated kinases (ERKs), is involved in transmitting signals from the cell membrane to the nucleus. The activation of the MAPK/ERK pathway can lead to the expression of genes involved in inflammation and cell proliferation, impacting the cytokine profile [35].

NF-κB is a key transcription factor that regulates the expression of many cytokines, including TNF-α, IL-6, and IL-10. Surface topographies that influence cellular stress and integrin signaling can lead to NF-κB activation, thus affecting cytokine expression [36].

In our next research stage, we plan to investigate these primary pathways to understand the surface topography-dependent alteration of cytokine production in CoP LPP.

The immunomodulatory properties of CoP LPP revealed in our study are of paramount importance. While numerous recent studies have explored the possibility of immune regulation at the wound site, most approaches involve incorporating bioactive compounds (such as growth factors, antibodies, etc.) into innovative formulations for wound-coating materials [22]. In contrast, our findings indicate that this prospective wound dressing material itself exhibits immunomodulatory properties, without additions.

Existing studies on novel wound dressing materials with inherent immunomodulatory properties typically focus on either pro-inflammatory or anti-inflammatory effects [22,23]. However, our data suggest that CoP LPP performs complex immunomodulation, finely tuning both pro- and anti-inflammatory processes at appropriate times. This characteristic of CoP LPP can be considered a significant advantage due to factors like cost-effectiveness and reduced risks compared to formulations containing human tissue-derived materials.

Despite these promising results, it is important to acknowledge the limitations of our study. While we plan to generally use LPPs as topical dressings, in our in vitro study, we are currently investigating the opposite: growing cells on top of LPP films instead of placing the LPP films on top of the cells. However, while this approach allows us to evaluate the promotion of adhesion, migration, proliferation, and cytokine production modulation, it may not fully replicate the in vivo environment. Therefore, future studies should include in vivo testing using mouse models.

Another limitation is that our research only used two cell types, without including keratinocytes, which are crucial for wound re-epithelialization. Conducting additional in vitro studies with keratinocytes will be necessary before proceeding to in vivo testing.

In summary, our results indicate that the application of novel biomimetic leucine-based pseudo-protein (LPP) films significantly improves wound closure rates, highlighting their potential as advanced wound dressing materials enhancing the wound healing process through superior cell support and dynamic cytokine regulation. Continued research and development in this area are essential to translate these findings into practical clinical applications, offering improved outcomes for patients with complex wound healing needs.

## 4. Materials and Methods

### 4.1. Reagents and Materials

Dulbecco’s Modified Eagle Medium (DMEM) (Sigma-Aldrich Chemicals, Cat. No. D5796, St. Louis, MO, USA), fetal bovine serum (FBS) (Capricorn Scientific GmbH, Cat No. FBS-HI-12A, Ebsdorfergrund, Germany), L-glutamine (Sigma-Aldrich Chemicals, Cat. No. G7513, St. Louis, MO, USA), paraformaldehyde (Carl Roth GmbH, Art.-Nr. 0335.1, Karlsruhe, Germany), 4′,6-diamidino-2-phenylindole (DAPI) (Cat. No. SC-3598), dimethyl sulfoxide (DMSO) (Cat. No. SC 202581A), phosphate-buffered saline (PBS) (Cat. No. SC-24947), and 2.5% Trypsin-EDTA (Cat. No. sc-391060) were all purchased from Santa Cruz Biotechnology Inc. (Dallas, TX, USA).

ELISA murine TNF-α (Cat. No. 900-K54), IL-6 (Cat. No. 900-K50), IL-10 capture kits (Cat. No. 900-K53), and an ABTS ELISA Buffer Kit (Cat. No. 900-K00) were all purchased from PeproTech (Cranbury, NJ, 08512, USA). The following monoclonal antibodies were all purchased from BioLegend (San Diego, CA, USA): anti-murine CD86-APC (Cat. No. 105011), CD206/MMR-PE (Cat. No. 141705), PE Rat IgG2a κ Isotype Ctrl Antibody (Cat. No. 400507), APC Rat IgG2a, and κ Isotype Ctrl Antibody (Cat. No. 400511).

Then, 35 mm Petri dishes with attached glass slides covered with collagen were purchased from MatTek Life Sciences (Cat. No. P35GCOL-1.5-14-C, Ashland, MA, USA).

The murine macrophage cell line RAW264.7 (ATTC TIB-71) was purchased from the American Type Cell Culture Collection (ATCC) (Manassas, VA, USA), while the primary mouse skin fibroblasts (pMSFs) had been previously prepared at our laboratory, as reported in [18].

Briefly, pMSFs were derived from the tails and ears of euthanized newborn mice (CD1 strain, Charles River Laboratories, Rowley, NC, USA). Prior to the procedure, all surgical instruments were sterilized. The tails and ears were cut and placed in a 50 mL polypropylene centrifuge tube, washing the tissue three times with 1× PBS with penicillin/streptomycin and amphotericin B. They were then minced into small pieces, and all samples were transferred to a new tube and incubated in 0.25% trypsin overnight at 4 °C. After incubation, the washing procedure was repeated. Then, the tissues were placed on a 10 cm Petri dish, and the skin layer was removed and minced into smaller pieces (less than 3 mm). The samples were washed in PBS by gentle shaking in a 50 mL polypropylene centrifuge tube. Afterwards, the samples were placed on 35 mm Petri dishes (10 small pieces in each), 2–3 drops of complete medium (DMEM with 10% FBS, 1% penicillin/streptomycin, and 2 mM L-glutamine) were added, and a sterile 20 mm glass coverslip was placed over the skin specimens. Afterwards, 2 mL of complete medium was added, and the cultures were incubated at 37 °C in a 5% CO_2_ atmosphere. The medium was changed every 2–3 days. After reaching 80% confluency, the coverslips were removed, and it became possible to passage the cells into T25 flasks. Fibroblast identification by detailed morphological analysis was performed using SEM and laser confocal microscopy after staining F-actin with phalloidin (Phalloidin iFluor 647, Abcam, Cambridge, UK) (results previously shown in [18]). Early passages (P1-P3) of the derived fibroblasts were cryopreserved at −86 °C for future experiments and used in the current study.

### 4.2. Synthesis of Leucine-Based PPs: 1L6, 8L6, and a Copolymer (1L6)_0.7_-(8L6)_0.3_

LPP synthesis was performed as described previously [18].

### 4.3. Preparation of Polymeric Films from LPPs 

A 7% solution of each LPP in absolute ethanol was prepared. Using 96-well plates, 40 µL of LPP solution was distributed per well. The wells were air-dried in a laminar flow hood. Then, using glass coverslips, each slip was covered with 100 µL of LPP solution and air-dried in a laminar flow hood.

### 4.4. Cell Cultures

Both cell types (pMSFs, RAW264.7) were cultured under standard conditions (+37 °C and 5% CO_2_) in DMEM supplemented with 10% fetal calf serum (FCS), 2 mM of L-glutamine, 50 U/mL of penicillin, and 50 μg/mL of streptomycin. At 80% confluence, the cells were harvested and seeded for the experiment on 96-well plates or coverslips either pre-coated with LPPs or not. The cells were seeded at the following concentrations: 25 × 10^4^ per mL of RAW264.7 cells and 15 × 10^4^ per mL of pMSFs.

### 4.5. In Vitro Wound Healing Assay

The pMSFs were grown on glass cover slides either untreated or pre-covered with LPP film until the confluent layer was achieved. The slides were then placed on 35 mm Petri dishes (Santa Cruz Biotechnology, Inc., Heidelberg, Germany). For the cells cultured on collagen-covered slides, commercially available 35 mm Petri dishes with attached collagen-covered glass slides were used (MatTek Life Sciences, Ashland, MA, USA). Cell motility was assessed by the wound healing assay [26,27]. Briefly, cell migration was initiated by removing a portion of the cell layer by scratching with a pippette (Eppendorf, Hamburg, Germany) tip (200 μL). The medium was changed to remove floating or damaged cells. The scratched samples were placed under a Zeiss LSM900 confocal microscope (Carl Zeiss AG, Oberkochen, Germany) equipped with a mini incubation system, and time-lapse imaging was performed every 10 min for 20 h using the inverted phase-contrast microscopy mode. Subsequently, a video was generated from the individually captured images using the Zen 3.0 software. The initial scratch width was measured with Zen 3.0, and each sample’s cell migration speed was calculated based on the scratch closure time and the initial distance between the edges.

### 4.6. Evaluation of Secreted Cytokine Levels

Cell culture supernatans after incubation (8 h, 24 h, and 72 h) were collected and frozen at −86 °C. Afterwards, the supernatants were assayed for their TNF-α, IL-6, and IL-10 levels by the sandwich enzyme-linked immunosorbent assay (ELISA) method, using commercially available murine TNF-α, IL-6, and IL-10 capture kits (PeproTech, Cranbery, NJ, USA) according to the manufacturer’s instructions. The ABTS ELISA Buffer Kit was used in conjunction with PeproTech’s Standard ELISA Development Kits.

### 4.7. Macrophage Phenotyping

After growing for 24 h in control conditions or on LPP films, the macrophages were phenotyped, according to the manufacturer’s standard protocol, using the following monoclonal antibodies: anti-murine CD86 and CD206/MMR (CD86-APC, CD206-PE, BioLegend, San Diego, CA, USA). Analyses of surface marker expression via flow cytometry were conducted after blocking unspecific binding sites with COHN fraction II (Sigma-Aldrich Chemicals, St. Louis, MO, USA). Appropriately matched and fluorescently conjugated isotype controls were also used. Flow cytometry was performed with a BD Accuri C6 (BD, Franklin Lakes, NJ, USA), and the data were processed with the related software version 1.0 (BD, Franklin Lakes, USA).

### 4.8. Statistical Analysis

The data are reported as the means ± standard errors of the mean (SEM) of at least three independent experiments. A Student’s *t*-test was used to perform a statistical analysis of the differences among the experimental groups, considering *p*-values < 0.05 statistically significant. In this study, all statistical analyses were performed using Microsoft Excel 2019.

## Data Availability

The data are available in a publicly accessible repository that does not issue DOIs. Publicly available datasets were analyzed in this study. These data can be found below: https://drive.google.com/drive/folders/1jrk974fYAqu9VLUzFVqpBbNtncnxL02h?usp=drive_link (accessed on 28 August 2024).

## Supplementary Materials

The following supporting information can be downloaded at https://drive.google.com/drive/folders/1wEShS9xUm-_8rHg86b7vWK4kNHt1mp8w?usp=drive_link (Accessed on 28 August 2024).
